# *β*-Thalassaemia Major in a Spanish Patient due to a Compound Heterozygosity for CD39 C → T/−28 A → C

**DOI:** 10.1155/2009/476342

**Published:** 2009-07-28

**Authors:** Soledad Gamarra, Guillermo Garcia-Effron, Carmen Monteserin, Isabel Lopez-Villar, Florinda Gilsanz, Joaquín Martinez-Lopez

**Affiliations:** ^1^Laboratorio de Biología Molecular, Servicio de Hematología, Hospital Universitario 12 de Octubre, 28041 Madrid, Spain; ^2^Public Health Research Institute, University of Medicine and Dentistry of New Jersey, 225 Warren Street, Room W210A, Newark, NJ 07103, USA; ^3^Servicio de Hematología, Hospital Universitario de Getafe, Carretera de Toledo s/n, Getafe, 28901 Madrid, Spain; ^4^Servicio de Hematología, Hospital Universitario 12 de Octubre, 28041 Madrid, Spain

## Abstract

A Spanish male patient with *β*-thalassaemia major was studied. Compound heterozygosity was found for one of the most common *β*-globin gene mutations in the Spanish population (codon 39 C → T) and for a mutation in the TATA box element of the *β*-globin gene promoter (−28 A → C mutation). To our knowledge this is the first report of a CD39 C → T and −28 A → C change association and the first report of the −28 A → C substitution in a Spanish patient.

## 1. Introduction

*β*-thalassaemia is a hereditary and heterogeneous group of disorders caused by mutations in the *β*-globin gene that result in the reduced or nonproduction of *β*-globin chains. The inheritance of one mutated allele is usually asymptomatic (*β*-thalassaemia trait), but the inheritance of two defective alleles (homozygote or compound heterozygote) would produce *β*-thalassaemia major (BTM) or intermedia (BTI) [1]. The severity of the symptoms of *β*-thalassaemia depends in part on the combination of *β*-globin gene mutations. However, predicting clinical phenotype based on the *β*-globin genotype is not always straightforward since there are other genetic factors than can ameliorate or worsen the *β*-thalassaemia phenotype [2–6]. Hence, clinical classification is based in the necessity to enroll a particular patient in a regular transfusion program (BTM) or not (BTI).

The mutations causing *β*-thalassaemia can be found in the *β*-globin gene exons, in the splice consensus sequences or in the transcription factor binding sites within the promoter region, resulting in a reduced efficiency of transcription initiation [1, 7]. Within each population, a small number of mutations are found. For example, in the Spanish population, a total of 86.6% of the alleles identified can be grouped into only five different mutations [8].

Here we describe a patient of Spanish descent with BTM (transfusion-dependent) who is compound heterozygous for one of the most common *β*-globin gene mutation in Spanish population (CD39 C → T) [8, 9] and an A to C substitution at position −28 relative to the transcription start site. This −28 mutation, first described in Kurdish Jew descendents, affects the TATA box element in the *β*-globin gene promoter reducing mRNA amounts [10, 11]. To our knowledge this is the first report of a CD39 C → T and −28 A → C mutations association and the first report of the −28 A → C substitution in a Spanish patient.

## 2. Materials and Patients

### 2.1. Case History

A Spanish male patient and his family were studied. The propositus is 21-year-old and was diagnosed to have *β*-thalassaemia major 20 years ago. He is transfusion-dependent each three weeks, to maintain a haemoglobin concentration ([Hb]) of 10 gr/dL. A blood count showed marked microcytic hypochromic anaemia with [Hb] 6.5 g/dL, mean corpuscular volume (MCV) of 64 fL and mean corpuscular haemoglobin (MCH) of 20 pg. Haemoglobin (Hb) analysis showed levels of HbA_2_ of 5.1% and HbF of 1.0%. In 1999 he was cholecystectomized due to an acute cholelithiasis, and in 2000 he was splenectomized. Actually he has been diagnosed of hemochromatosis due to iron overload, and he is in a program of iron chelation with desferrioxamine. Both parents are Spanish and both showed a thalassaemia minor phenotype. The mother showed minimal anaemia ([Hb] 11.6 g/dL) with slightly hypochromic and microcytic (MCV 69 fL, MCH 21.8 pg) and increased HbA_2 _ 4.8% and HbF 1.9%. The father showed milder anaemia with [Hb] 13.4 g/dL, MCV 71 fL, MCH 23 pg, HbA_2 _ 5.6%, and HbF 1.0%.

### 2.2. DNA Isolation

DNA was extracted from peripheral EDTA anticoagulated whole blood using the MagNA Pure LC automated system (Roche Applied Science, Manheim, Germany) following the manufacturer's instructions.

### 2.3. *β*-Globin Gene Analysis

The propositus and his parents were studied for the most frequent *β*-thalassaemia mutations in the Mediterranean area by procedures based upon real-time PCR and specific fluorescent labeled hybridization probes including: IVSI-II-745 C → G, CD5 (-CT), CD6 (-A), CD8 (-AA), CD39 C → T, CD37 G → A, IVSI-I G → A, IVSI-6 T → C, and IVSI-110 G → A [12, 13]. The full coding, the 5′UTR, and the 3′UTR sequences of *β*-globin gene (GenBank accession no. U01317) were amplified and sequenced to discard other mutations. Primers sequences are displayed in Table 1. The amplifications were performed in a 25 *μ*L volume, containing 25 mM Cl_2_Mg, 200 *μ*M of each dNTP (Promega, Madrid, Spain), 2.5 U Expand Long Template PCR System (Roche), 0.6 *μ*M each primer, and 100 ng DNA. Amplification was performed in a PTC-100 thermal cycler (MJ Research INC, Madrid, Spain) for one cycle of 10 minutes at 95°C and then for 35 cycles of 20 seconds at 95°C, 20 seconds at 62°C, and 1 minute at 72°C, followed by one final cycle similar to the previous one but with 10 minutes at 72°C. The PCR products were analyzed by electrophoresis.

### 2.4. DNA Sequencing

PCR products were purified using ExoSAP-IT (GE Healthcare, Little Chalfont, UK). The PCR fragments were sequenced by the BigDye terminator V3.1 Cycle Sequencing ready reaction system (Applied Biosystems, Ca, USA) according to the manufacturer's instructions. Sequencing primers are displayed in Table 1. Sequence analysis was performed on an ABI Prism 3100-Avant DNA sequencer (Applied Biosystems).

### 2.5. Analysis of *α*-Globin Genes as Secondary Modifiers of the *β*-Thalassemia Clinical Phenotype

*β*-thalassemia clinical manifestations could be alter by concomitant *α*-thalassemia or by extra copies of the *α*-globin genes [6, 14]. In order to evaluate this fact, the most frequent Mediterranean *α*-thalassemia deletions (−*α* ^3.7^, −*α* ^4.2^, --^MED^) and the presence of extra copies of the *α*-globin gene were determined in the proband and in his parents by PCR-GAP [15].

## 3. Results and Discussion

We have studied a Spanish patient with a BTM phenotype due to a compound heterozygous genotype never published before, a *β*-globin exon gene mutation (C → T transition at CD39 position) and a *β*-globin TATA box mutation (A → C mutation in the −28 position). The first nucleotide substitution was inherited paternally while the TATA box mutation was inherited maternally. The CD39 C → T mutation is one of the most common mutations in Mediterranean countries causing up to 64% of all the Spanish *β*-thalassaemias [9]. On the other hand, the −28 A → C mutation had been barely described in the scientific literature. The first report describing this allele was published in 1982 where two Kurdish Jews siblings with BTM were studied [10]. In a follow-up paper, the same group established that the patients were compound heterozygotes for this TATA box mutation [11]. In 1992, Basak et al. [16] studied the mutations in the *β*-globin gene in a group of Turkish patients exhibiting BTI and BTM. In this population the −28 A → C mutation was rare but the authors did not establish the genotype (homozygous or compound heterozygous) or the phenotype linked with the mutation. In 1996, Perea et al. [17] described a Mexican mestizo family with BTM due to a −28 A → C mutation and a CD11 –T frame shift compound heterozygosity. The TATA box mutation in this family was linked to the same haplotype as described previously for the Kurdish Jews siblings [11]. In 2005, two molecular epidemiological reports were published studying Middle East populations where the −28 A → C mutation appears to be a rare allele. Adekile et al. [14] described an Iraqi patient who was a compound heterozygote for the −28 A → C and the IVS-II-1 G → A mutations. This patient showed a BTI phenotype. The mild clinical manifestation was linked to a −158 C → T polymorphism of the *γ*-globin gene promoter with elevated Hb F together with *α*-gene deletion. In the same year, Darwish et al. [18] report 1 patient harboring the −28 A → C mutation in homozygosis out of 148 Palestine *β*-thalassemia patients studied. Unfortunately, the phenotype of this patient was not mentioned. Turning to our patient, the BTM phenotype is consistent with the clinical manifestation observed in the other compound heterozygous patients described so far. Moreover, our patient and his family showed no *α*-thalassemia deletions (−*α* ^3.7^, −*α* ^4.2^, --^MED^), extra copies of *α*-globin genes, or HbF elevation. Thus, the propositus BTM phenotype could be related strictly to the *β*-globin mutations described here.

## Figures and Tables

**Table 1:** Oligonucleotide primers used in this work.

Primers	5′ → 3′	Sequence (5′ → 3′)	Gene	Use
BF1	Sense	TCCAGGCAGAAACAGTTAGATG	*β*-Globin	Amplification and Sequencing
BF2	Sense	GAAGAGCCAAGGACAGGTAC	*β*-Globin	Sequencing
BF3	Sense	TGGCTCACCTGGACAACCTC	*β*-Globin	Sequencing
BF4	Sense	TCAGGGCAATAATGATACAA	*β*-Globin	Sequencing
BR1	Antisense	ATGCACTGACCTCCCACATTC	*β*-Globin	Sequencing
BR2	Antisense	CCAGCCTTATCCCAACCATAAA	*β*-Globin	Sequencing
BR3	Antisense	TCACAGTGCAGCTCACTCAGT	*β*-Globin	Sequencing
BR4	Antisense	CAACTTCATCCACGTTCACC	*β*-Globin	Amplification and Sequencing
